# Ocean acidification promotes otolith growth and calcite deposition in gilthead sea bream (*Sparus aurata*) larvae

**DOI:** 10.1038/s41598-018-26026-y

**Published:** 2018-05-30

**Authors:** Clara Coll-Lladó, Jan Giebichenstein, Paul B. Webb, Christopher R. Bridges, Daniel Garcia de la serrana

**Affiliations:** 1 Gatty Marine Laboratory, Scottish Oceans Institute, School of Biology, University of St Andrews, Scotland, UK; 2 Xelect Ltd, Horizon House, St Andrews, Scotland, UK; 3 Institut für Stoffwechselphysiologie/AG Ecophysiology, Heinrich-Heine-Universität, Düsseldorf, Germany; 4 School of Chemistry, University of St Andrews, Scotland, UK

## Abstract

The effects of ocean acidification on otolith crystallization and growth rates were investigated in gilthead sea bream (*Sparus aurata*) larvae. Larvae were exposed to three different pH levels: pH8.2, pH7.7 and pH7.3 for a period of 18 days post-fertilization. For the first time, we demonstrate that pH has a significant impact on the carbonate polymorph composition, showing calcite in a significant percentage of individuals at low pH. Around 21% of the larvae exposed to pH7.3 showed irregular calcitic otoliths rather than commonly found round aragonitic otoliths. Calcitic otoliths showed a moderate level of heritability suggesting an important role of genetic factors. We also observed significantly larger otoliths in larvae reared at pH7.7 and pH7.3 compared to pH8.2 in both sagittae and lapilli. Our results demonstrate that otolith growth rates in gilthead sea bream larvae increase at low pH while a significant proportion of larvae are prone to the formation of calcitic otoliths at pH7.3.

## Introduction

Ocean acidification is a major consequence of rising levels of atmospheric carbon dioxide (CO_2_). The average ocean surface *p*CO_2_ has risen from pre-industrial values around 280 µatm to the current 390 µatm and is estimated to reach 1000 µatm and 1900 µatm by 2100 and 2300^1^ respectively; this is equivalent to a decline of 0.32 and 0.77 units in the average ocean surface pH^1^.

The impact of ocean acidification on marine ecosystems has been the focus of intensive research^2^. This pH decline is predicted to affect the saturation states of the different calcium carbonate (CaCO_3_) polymorphs (aragonite, calcite and vaterite)^3^ altering the formation and dissolution rates of carbonate-based structures in marine organisms including shells and exoskeletons^4^. Several studies have investigated the effects of ocean acidification on biomineralization in groups such as molluscs, echinoderms, corals, crustaceans, sponges and dinoflagellates^4^. The potential impact of ocean acidification on fish has also been studied, but relatively less compared to invertebrate groups^5^. Studies on fish have focused on acid-base regulatory capacity^6^, growth^7^, development^8^, neurosensory functions and behaviour^9^, reproduction^10^, metabolism^11^ and otolith formation^12^.

Otoliths, commonly called earstones, are calcified structures located in the fish inner ear and are the organs responsible for sensing gravity, balance, linear acceleration and sound. Fish normally have three pairs of otoliths: the largest sagittae, the lapilli and the smallest asterisci. They are formed by CaCO_3_ crystals embedded in a non-collagenous organic matrix composed of acidic proteins and polysaccharides^13–15^. Otoliths are often used as a tool for age determination^16^, owing to the visible growth rings of different density (indicating from daily to seasonal increases) and for the study of fish life histories and environmental effecs^17,18^. In almost all teleost fish the sagittae and lapilli are composed of aragonite while the smaller asterisci can be composed of vaterite^19,20^. Vateritic sagittae and lapilli are present in some species of non-teleost fish^21^, and can be rarely found in some individuals from wild populations and in a significant proportion of aquaculture-reared animals^22–27^. Otolith formation in larvae starts with precursor spherules in the otic cavity where calcium carbonate (CaCO_3_) precipitates^28^; just after 30 hours post-fertilization (hpf) a mineralized ovoid is already visible^29^. Otolith formation and growth is determined by the conditions and composition of the surrounding endolymph (fluid^30^, flow^31^ and pH^32–34^), temperature^35^, metabolism^36^ and composition of the organic matrix^37^. Endolymph is more alkaline (pH = 8.0) compared to plasma (pH = 7.7), partly as consequence of the bicarbonate content and a relatively higher concentration of CO_2_ (32 mmol^−1^) compared to plasma (14 mmol^−1^)^33^. Several studies have showed that low environmental pH increases otolith growth rate in some species of fish^26,38–40^. Some studies have suggested that hearing capacity and balance are a function of otolith size, shape and structure^39–41^. Thus, the impact of higher otolith growth rates due to lower oceans’ pH in fish ecophysiology could be significant.

The Mediterranean Sea, with its limited water exchange with the Atlantic Ocean at the West and the Black Sea to the East, is predicted to be especially sensitive to ocean acidification^42^. The impact of the pH decline on otolith formation in Mediterranean fish is restricted to a single study on gilthead sea bream (*Sparus aurata*) juveniles^43^. To increase our knowledge of ocean acidification impact on otolith formation in Mediterranean fish we studied how different levels of *p*CO_2_ affect otolith growth during the first stages of gilthead sea bream development. At these early stages of development fish are more vulnerable to environmental factors due to their reduced acclimation capacity and limited ability to avoid unfavourable conditions. The effects of otolith formation in gilthead sea bream were studied following exposure to three different levels of *p*CO_2_ with corresponding changes in pH: 287 µatm (pH8.2; pre-industrial levels), 1159 µatm (pH7.7) and 2650 µatm (pH7.3) for 18 days after egg fertilization.

## Methods

### Experimental conditions

All experiments were performed at the Malta Aquaculture Research Centre (MARC), Fort Torri San Luċjan Marsaxlokk (Malta), part of the Directorate for Aquaculture of the Maltese Government. All protocols were approved by the Directorate of Aquaculture of the Ministry for the Environment, Sustainable Development and Climate Change for the growth of larval fish in a commercial hatchery in accordance with all relevant guidelines and regulations (EU Directive 2010/63/EU). This is a registered facility for Aquaculture Production in Malta and complies with all statutory regulations for the production of Aquaculture Fish. The fish larvae used in the present study were taken from the production facility and kept in comparable conditions as found in normal fish cultivation. Larvae were euthanized and samples were taken following the standard protocol procedure from the MARC.

Gilthead sea bream (*Sparus aurata*) eggs were obtained from a brood stock kept in the Aquaculture Directorate facilities at normal seawater pH levels in 2016. The eggs were produced in two independent batches (mass spawning events) using the same set of parents; the first batch was used for the pH8.2 and pH7.7 groups while the second batch was used for the pH7.3 group. Fertilized eggs were collected with a net system in the overflow from a 14 m³ brood stock tank containing 50 individuals. The freshly collected fertilized eggs were moved to the experimental tanks (8 tanks per experimental condition) consisting of 80 L fibreglass tanks, which were stocked with 200 eggs/L in a flow-through system. To obtain similar conditions in the tanks of the same treatment, a 60 L header-tank with a temperature control unit (IKS Aquastar Computer Systeme GmbH, Germany; Titanium Heater, Aqua Medic 500 W) was connected to the water inflow of each tank. From the header tank, each experimental tank was fed with 150 ml of water/min. Each treatment with its own header tank was supplied with a controlled amount of CO_2_, which originated from a gas bottle directly connected to the header tanks via a valve. The CO_2_ influx was regulated by a magnetic valve (M-ventil Standard, Aqua Medic GmbH, Germany) while pH was controlled by an IKS-system (IKS Aquastar Computer Systeme GmbH, Germany) and monitored with pH electrodes (pH 3310, WTW, Germany). Three different pH levels were used: pH8.2 (control) and two experimental conditions at pH7.7 and pH7.3. Water temperature was maintained between 17–18 °C for all treatments. Embryos were kept in darkness until 6 days post-fertilization (dpf) and afterwards kept on 12 h light and 12 h dark (07:30–19:30) regime. From 6 dpf the hatched larvae were held in a green water system (Nanochloropsis, Nanno3600, Reed Mariculture Inc, USA) and were fed three times per day (09:30; 14:00; 18:30) with enriched rotifers (Red Pepper, BERNAQUA, Belgium). Water alkalinity (Palintest, Alkaphot) and oxygen saturation (Handy Polaris, OxyGuard®) were monitored during the entire experiment (Table 1 and Supplementary Figure 1). Animals were exposed to the three different pH treatments for 18 dpf in total and after that period an average of 500–1000 larvae per tank were randomly sampled and preserved in 70% ethanol for further analysis.

**Table 1:** Gilthead sea bream rearing conditions and otolith morphometry.

	pH8.2	pH7.7	pH7.3
*p*CO_2_ (µatm)	287 ± 7.65	1159 ± 119	2650 ± 208
CaCO_3_ (mg/l)	28.4 ± 7.6	28.4 ± 7.6	24.4 ± 6.9
O_2_ (mg/l):%	6.98 ± 0.28:92	6.97 ± 0.27:92	7.08 ± 0.32:93
Temperature (°C)	17.3 ± 0.1	17.3 ± 0.1	17.5 ± 0.3
Number of individuals analysed	111	174	186
Larval total length (mm)	3.6 ± 0.4	3.7 ± 0.4	3.6 ± 0.4
Sagittae (ρ_S_) correlation with larval length	0.60	0.40	0.53
Lapilli (ρ_L_) correlation with larval length	0.40	0.32	0.42
Sagittae *OA* (µm^2^)	736 ± 128	793 ± 118	947 ± 162
Sagittae *OP* (µm)	99 ± 9	104 ± 30	121 ± 11
Lapilli *OA* (µm^2^)	500 ± 101	570 ± 93	675 ± 105
Lapilli *OP* (µm)	81 ± 8.5	86 ± 7.5	94 ± 8.5
Normalized sagittae *OA*^#^	201 ± 28	214 ± 30	260 ± 38
Normalized lapilli *OA*^#^	136 ± 25	154 ± 25	185 ± 27
Normalized sagittae *OP*^#^	27 ± 2	29 ± 2	31 ± 2
Normalized lapilli^#^	22 ± 2	24 ± 3	26 ± 3
Sagittae Form Factor (*FF*)^†^	0.93 ± 0.04	0.94 ± 0.13	0.93 ± 0.07
Lapilli Form Factor (*FF*)^†^	0.93 ± 0.04	0.95 ± 0.02	0.94 ± 0.04
Calcitic otoliths (%)	0	1.2 ± 3.1	21.1 ± 10.5

All values are expressed as mean ± standard deviation. Water parameters were averaged for all replicate tanks and days of treatment, therefore represent global treatment values.

ρ_S/L_ values indicate Pearson correlation coefficient.

^#^Normalized values = *OA* or *OP*/Larval total length.

^†^Form Factor (FF) = 4π*OA*x*OP*^−2^, where *OA* is the sagittal area and *OP* is the perimeter.

Calcitic = percentage of larvae with at least one of the two otolith pairs formed by calcite.

### Otolith removal and measurements

A minimum of 12 larvae per treatment tank were randomly selected and individually photographed for body measurements using an AxioCam Zeiss camera attached to a Leica Wild M3C stereo microscope using AxioVision Rel v.4.8 software and a millimetre scale for calibration. Larval total length was measured using ImageJ.

For *in situ* observation of the otoliths’ general morphology, larval heads were photographed and digitalized using a Leica DFC320 inverted microscope and Leica Application Suite v.2.5.0 software. In order to take accurate measurements of the otoliths the whole brain was extracted under a stereo microscope using two micro dissecting needles, individually placed in 96-well plates and soft tissue was digested with a 1% sodium hypochlorite solution (Sigma, Dorset, UK) at 4 °C overnight. After the digestion, otoliths were at the same level in the bottom of the well and were digitalized. Extracted and digested otoliths were photographed and digitalized using a Leica DFC320 inverted microscope and Leica Application Suite v.2.5.0 software. Otolith area (*OA*) and perimeter (*OP*) were measured with ImageJ using the microscope internal scale as reference. Measurements were done twice to ensure that all measurements were accurate. *OA* and *OP* from both the left and right otoliths were averaged, distinguishing between the bigger (predicted sagittae) and smaller (predicted lapilli) otoliths (Supplementary Figure 2). The asterisci were not observed. The normalized *OA* and *OP* were calculated dividing the *OA* and *OP* values for sagittae and lapilli by the animal total body length. The form factor (*FF*) index was estimated as *4πOAxOP*^*−*2^. Unless indicated otherwise, all values for those parameters are provided as mean ± SD.

Extracted and digested otoliths were further categorized as “round” or “irregular” based on their external appearance under the microscope. Those with clearly round and smooth surfaces were categorized as “round” otoliths while those with angular and polygonal surfaces were categorized as “irregular” otoliths. This nomenclature was used until the main calcium carbonate polymorph forming the round and irregular otoliths was determined. For calcium carbonate polymorph determination otoliths were washed several times with milliQ water to remove any remnants of sodium hypochlorite. Then, they were placed on a borosilicate plate and dried overnight at 35 °C.

### Raman spectroscopy

The otolith calcium carbonate polymorph composition was determined using Raman spectroscopy. Raman spectra were recorded from 5 round and 5 irregular otoliths with a Horiba Jobin Yvon LabRam HR instrument using 514 nm excitation and a 50× magnification long-working distance objective. Laser intensity was attenuated using neutral density filters to ensure that laser-induced transformation of the polymorph was not occurring. Spectra were recorded from the centre of the otoliths. Aragonite, calcite and vaterite minerals (kindly provided by Dr Nicola Allison, School of Earth and Environmental Sciences, University of St Andrews) were also recorded as standards to correctly identify the CaCO_3_ polymorph present in the larval otoliths.

### Genotyping and pedigree reconstruction

A total of 50 larvae exposed to pH8.2, 50 exposed to pH7.7, 100 larvae exposed to pH7.3 showing aragonitic otoliths and 100 larvae exposed to pH7.3 that had calcitic otoliths were genotyped. Genotyping was carried out by the biotechnology company Xelect Ltd using a 95 single nucleotide polymorphism (SNP) panel designed for gilthead sea bream pedigree assignment and validated following quality filters previously described for SNPs^44^. Larval DNA was extracted using a 10% (w/v) chelex 100 (Sigma) solution containing 2% proteinase K (20 mg/ml; Sigma) for 1 h at 55 °C followed by an inactivation step at 95 °C for 10 min.

DNA samples were genotyped using a Fluidigm BioMark platform following the manufacturer recommendations. Prior to genotyping, samples were submitted to 14–16 cycles of pre-amplification in order to increase DNA concentration. A Fluidigm 96 × 96 chip was loaded with the pre-amplified samples diluted 1:50 (v/v) in nuclease-free water and 95 SNP assays labelled with FAM and HEX-probes. The 96 × 96 v2 PCR program was selected following the manufacturer recommendations. After completion, the relative fluorescence of the labelled probes was read in the Fluidigm EP1 reader using EP1 Data collection software v3.1.1. Larval individual genotypes were called using the Fluidigm SNP Genotyping Analysis software.

Genotypes were employed for pedigree reconstruction using the Colony software package^45^ and the following parameters were selected: diploid species with separate sexes, polygamic mating system, 3 medium length runs, full-likelihood (FL) analysis method, update allele frequencies, assume no prior for the sibship prior option and default random number of seeds. Pedigree reconstruction was performed using two different levels of SNP dropout rate (10 or 20%) and, since we did not have accurate estimates of the parents inbreeding level, models were constructed considering either a significant or not significant level of inbreeding. Using this criteria, we obtained four different pedigree reconstruction models: 20% SNP dropout with parental inbreeding (model A), 20% SNP dropout without parental inbreeding (model B), 10% SNP dropout with parental inbreeding (model C) and 10% SNP dropout without parental inbreeding (model D).

### Statistical analyses

All statistical analyses were conducted using R-Studio v.1.1.419^46^. The correlation between larval total length (*length*) and *OA* and *OP* parameters in response to pH treatments (*treatment*) were analysed using linear mixed models (*lme4* R-package)^47^ with *length* and *treatment* as fixed factors while *tank* and *batch* were introduced as random factors in order to control for their effects. Normalized *OA* and *OP* were also analysed using a linear mixed model with *treatment* as fixed factor and *tank* and *batch* as random factors. All mixed model analyses were followed by a Tukey-HSD *post-hoc* correction for pairwise comparisons (*lmerTest*)^47^. Differences were considered significant when p < 0.05.

Narrow-sense heritability (*h*^2^) for calcitic otoliths was estimated using a general linear mixed model (glmm). The Bayesian Markov chain Monte Carlo-based MCMCglmm R package^48^ was used with the results obtained from the Colony pedigree reconstruction as input and using a *categorical* distribution. For the MCMCglmm model construction we considered *treatment* as fixed factor, and half-sib *family* and *batch* as random factors. The average *h*^2^ value and confidence intervals were estimated using the following formula: *h*^2^ < - model$VCV[, “random factors”]/(model$VCV[, “random factors”] + model $VCV[, “units”] + 1). Mean *h*^2^ and 95% confidence intervals were calculated for each of the Colony models used.

All graphs were produced using the ggplot2 R-build package. R-regression plots include 95% confidence intervals estimated using the *geom_smooth (method* = *“lm”)* options.

## Results

### Otolith dimensions

Two pairs of otoliths were observed on each side of the fish head behind the eyes (Supplementary Figure 2). The most posterior otolith pair (the sagittae) were consistently larger than the most anterior pair (the lapilli) (Supplementary Figure 2), while the asterisci were not observed. The otoliths’ area (*OA*), otoliths’ perimeter (*OP*) and the larval body length were measured for a total of 111 (13–14 individuals per tank), 174 (23–26 individuals per tank) and 186 (25–30 individuals per tank) larvae exposed to water at pH8.2, pH7.7 and pH7.3, respectively.

**Figure 1:**
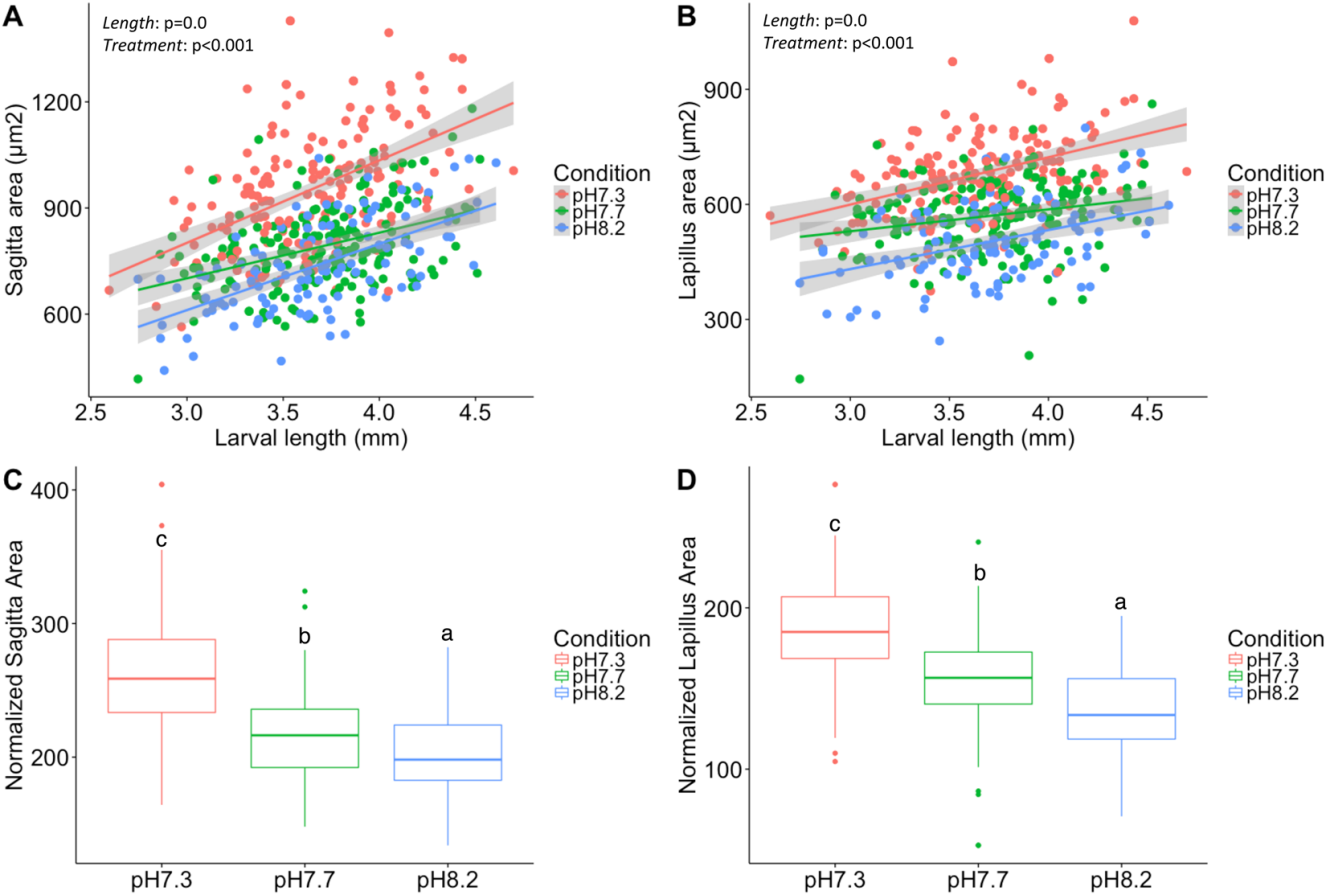
Correlation between otolith average area and larval body length in response to pH treatments. Larval total body length plotted against average sagittal (**A**) and lapillus (**B**) areas from animals exposed to pH7.3 (red circles), pH7.7 (green circles) and pH8.2 (blue circles). Correlations are indicated as red (pH7.3), green (pH7.7) and blue (pH8.2) lines; 95% confidence intervals are highlighted in grey around the linear correlation. Boxplot of normalized sagittal area (**C**) and normalized lapillus area (**D**) for pH7.3 (red box), pH7.7 (green box) and pH8.2 (blue box). Level of significance for pH *treatment* and animal *length* factors are indicated for each correlation. Different letters indicate significant differences between treatments (p < 0.05).

We did not find significant differences in larval length (p = 0.24) between treatments, suggesting that low pH had no impact on body growth rate. However, we found a strong positive correlation between larval body length and both sagittae and lapilli *OA* and *OP* (p < 0.001) (Tables 1–2; Fig. 1A,B). We also found that the pH treatment had a significant impact on the average *OA* (p < 0.001) and *OP* (p < 0.001) for both sagittae and lapilli. Larvae reared at pH7.7 (*OA* = 793 ± 118 µm^2^; *OP* = 104 ± 30 µm) and 7.3 (*OA* = 947 ± 162 µm^2^; *OP* = 121 ± 11 µm) had larger sagittae compared to those reared at pH8.2 (*OA* = 736 ± 128 µm^2^; *OP* = 99 ± 9 µm) with similar results observed in lapilli from pH7.7 (*OA* = 570 ± 93 µm^2^; *OP* = 86 ± 7 µm) and pH7.3 (*OA* = 675 ± 105 µm^2^; *OP* = 94 ± 8 µm) compared to pH8.2 (*OA* = 500 ± 101 µm^2^; *OP* = 81.8 µm) (Tables 1 and 2; Fig. 1A,B). Pairwise comparisons between pH8.2 and pH7.3 showed that differences were highly significant (p < 0.001; Table 2) for both sagittae and lapilli, while pH8.2 and pH7.7 differences were strongly significant for lapilli *OA* and *OP* (p < 0.001; Table 2) and sagittae *OP* (p = 0.008), but only marginal in the case of sagittae *OA* (p = 0.049; Table 2).

**Table 2:** Level of significance between treatments for the otolith parameters measured.

Parameter	Linear Mixed Model		Tukey Post Hoc	
	Factors	Par. P-value		
Sagittae *OA* (µm^2^)	*Length*	<0.001	*pH7.7 vs pH7.3*	<0.001
	*Treatment*	<0.001	*pH8.2 vs pH7.3*	<0.001
			*pH8.2 vs pH7.7*	0.051
Lapilli *OA* (µm^2^)	*Length*	<0.001	*pH7.7 vs pH7.3*	<0.001
	*Treatment*	<0.001	*pH8.2 vs pH7.3*	<0.001
			*pH8.2 vs pH7.7*	<0.001
Sagittae *OP* (µm)	*Length*	<0.001	*pH7.7 vs pH7.3*	<0.001
	*Treatment*	<0.001	*pH8.2 vs pH7.3*	<0.001
			*pH8.2 vs pH7.7*	0.008
Lapilli *OP* (µm)	*Length*	<0.001	*pH7.7 vs pH7.3*	<0.001
	*Treatment*	<0.001	*pH8.2 vs pH7.3*	<0.001
			*pH8.2 vs pH7.7*	<0.001
Sagittae Normalized OA*	*Treatment*	<0.001	*pH7.7 vs pH7.3*	<0.001
			*pH8.2 vs pH7.3*	<0.001
			*pH8.2 vs pH7.7*	0.042
Lapilli Normalized OA*	*Treatment*	<0.001	*pH7.7 vs pH7.3*	<0.001
			*pH8.2 vs pH7.3*	<0.001
			*pH8.2 vs pH7.7*	0.001
Sagittae Normalized OP*	*Treatment*	<0.001	*pH7.7 vs pH7.3*	<0.001
			*pH8.2 vs pH7.3*	<0.001
			*pH8.2 vs pH7.7*	0.032
Lapilli Normalized OP*	*Treatment*	<0.001	*pH7.7 vs pH7.3*	<0.001
			*pH8.2 vs pH7.3*	<0.001
			*pH8.2 vs pH7.7*	<0.001
Form Factor (*FF*)^#^	*Treatment*	0.10	*pH7.7 vs pH7.3*	0.07
			*pH8.2 vs pH7.3*	0.83
			*pH8.2 vs pH7.7*	0.38

*OA* = Otolith area.

*OP* = Otolith perimeter.

^*^Normalized values = *OA* or *OP*/animal length.

^#^Form Factor (FF) = 4π*OA*x*OP*^−2^, where OA is the sagittal area and OP is the perimeter.

When normalized data was compared we found an increase of 7% and 23% and 12% and 25% on sagittae and lapilli normalized *OA* in animals reared at pH7.7 and pH7.3 when compared to the pH8.2 group with similar differences for *OP* (Tables 1 and 2).

No differences in the form factor (*FF*) index were found between the otoliths of larvae reared at pH8.2 compared to pH7.7 or pH7.3 (Tables 1–2).

### Otolith morphology and calcium carbonate composition

**Figure 2:**
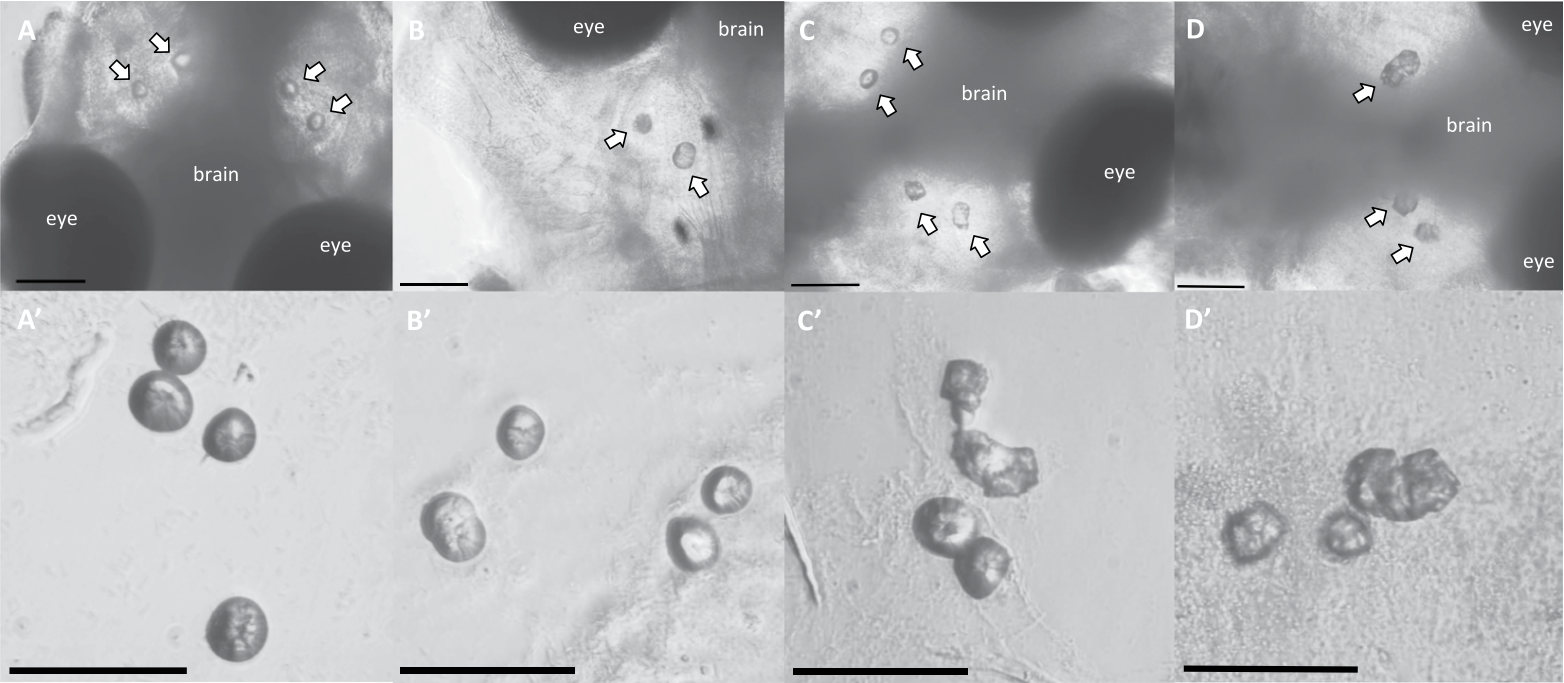
Gilthead sea bream larval otolith morphology. General (**A**–**D**) and detailed (**A’-D’**) views of round (**A,B**) and irregular (**C**,**D**) otoliths in gilthead sea bream larvae exposed to pH7.3. Otoliths inside the larval heads are indicated with white arrows. Scale bars indicate 100 µm.

While the majority of otoliths observed were round in shape regardless of the treatment (Fig. 2A,B), some larvae were found to have irregularly shaped otoliths, affecting both the sagitta and lapillus of either one or both head hemispheres (Fig. 2C,D). While irregular otoliths were absent in individuals reared at pH8.2, an average 1.2% and a staggering 21% of larvae reared at pH7.7 and pH7.3 (Table 1) were found to have irregular otoliths. Irregular otoliths had a similar average area than the round otoliths found in animals reared at pH7.3 (p = 0.56 and p = 0.41 for the sagittae and lapilli *OA*, respectively) (Supplementary Figure 3), but had a significantly lower FF index (*FF* = 0.87; p < 0.01) (Supplementary Figure 3) compared to round otoliths from any of the other groups. Interestingly we also found a few cases (N = 10) in which two otoliths seemed to be fused into a single unit (Fig. 2C,D).

**Figure 3:**
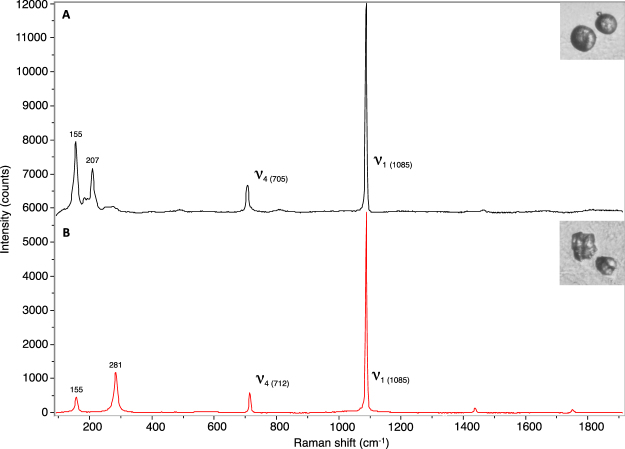
Raman spectroscopy of round and irregular otoliths. Raman spectroscopy profiles for aragonitic round otoliths (**A**) and calcitic irregular otoliths (**B**) in gilthead sea bream larvae exposed to pH7.3. Typical calcium carbonate ν_1_ and ν_4_ peaks are indicated and the shift position of each peak is indicated in brackets. Representative round and irregular otoliths are shown for each spectrum. Spectrums are showed separately in order to facilitate the visualization of the different peaks.

The CaCO_3_ polymorph composition in round and irregular otoliths was analysed using Raman spectroscopy (Fig. 3). Round and irregular otoliths displayed characteristic Raman shifts (1085 cm^−1^, 705–712 cm^−1^ and 155 cm^−1^) corresponding to the ν_1_ and ν_4_ vibrational modes of the CaCO_3_ lattice (Fig. 3). Round otoliths also exhibited a peak at 207 cm^−1^ (Fig. 3A) whilst irregular otoliths exhibited a peak at 281 cm^−1^ (Fig. 3B) corresponding to aragonite and calcite profiles respectively. This is similar to the profiles observed in previous studies of CaCO_3_ biomineralization^49^. The otolith composition was further confirmed by the Raman profiles of aragonite and calcite mineral standards (data not shown). These results clearly indicate that round otoliths were composed of aragonite whereas irregular otoliths were made of calcite.

### Pedigree reconstruction and heritability estimation

To estimate the proportion of variation attributable to genetic factors in the formation of calcitic otoliths, and rule out any *batch* effect in the calcitic phenotype, we performed a pedigree reconstruction for the gilthead sea bream larvae exposed to the three-different pH regimes and estimated the narrow-sense of heritability for the trait (*h*^2^). Four different pedigrees were reconstructed assuming the presence or absence of brood stock inbreeding and a 10% or 20% of SNP dropout (see Methods). Models A (assuming parental inbreeding and 10% dropouts) and B (without parental inbreeding and 10% dropouts) included a total of 247 individuals (48 from pH8.2, 42 from pH7.7, 80 from pH7.3 with aragonitic otoliths and 77 from pH7.3 with calcitic otoliths). Models C (assuming inbreeding and 20% dropouts) and D (without inbreeding and 20% dropouts) included a total of 234 individuals (48 from pH8.2, 42 from pH7.7, 78 from pH7.3 with aragonitic otoliths and 66 from pH7.3 with calcitic otoliths). All models identified more than 20 half-sib families (i.e. offspring that has one parent in common): models A and B comprised a total of 50 and 45 half-sib families respectively formed by 2 to 16 offspring per family, and models C and D comprised 45 and 26 half-sib families respectively, formed by 2 to 43 offspring per family (Supplementary Figure 4).

The majority of half-sib families with more than 3 offspring were formed by a mixture of larvae from treatments pH8.2, pH7.7 and pH7.3 (including aragonitic and calcitic otoliths) (Supplementary File 4). Only some half-sib families formed by 2 or 3 offspring appeared to be formed by individuals from a single treatment (normally from pH7.3), likely due to the over-representation of individuals from pH7.3 treatment compared to pH8.2 and pH7.7 (Supplementary Figure 4). The family structure inferred from all models indicate that the same group of parents (or at least a significant proportion of them) contributed to both spawning events, indicating that the batch did not have a significant effect on the differences observed between groups.

The reconstructed pedigrees from the four models were used to calculate the narrow-sense heritability (*h*^2^) for the calcitic otolith phenotype. All models showed a moderate level of *h*^2^ ranging from 0.44 to 0.55 on average depending on the model, with a 95% confidence interval expanding from 0.21 to 0.76 (Supplementary Figure 4), suggesting a significant contribution of genetic factors to the observed variation of the phenotype.

## Discussion

We show that otolith growth in gilthead sea bream larvae significantly increased when exposed to low pH (pH 7.7 and pH 7.3 compared to controls at pH8.2) and that 21% of the larvae precipitated their otoliths in calcite at pH7.3 instead of aragonite. Previous studies examining the potential effects of ocean acidification on fish otoliths have also shown increased otolith size^38,43^. However, this is the first time that calcitic otoliths have been observed as a consequence of exposure to low pH in fish larvae.

The levels of *p*CO_2_ used in the present study did not have a significant effect on larval growth rate, as suggested by the absence of differences in larval length between pH treatments, which indicates that gilthead sea bream larvae are effective acid-base regulators, like other fish species reared in similar conditions^50,51^. Otolith size strongly correlated with fish body length, as expected based on previous studies^43,52^. Despite not affecting larval somatic growth, low pH had a significant impact on otolith growth rate when larvae were exposed to pH7.7 and pH7.3 (Tables 1 and 2). The increase in larval otolith growth rate has already been reported for some species when exposed to elevated *p*CO_2_ levels^38,40^. However, other species have not shown such effects^7,50,51^, suggesting that it might be either a species-specific phenomenon or due to differences in experimental setups. This is the first study showing that gilthead sea bream, a characteristic Mediterranean species and a key aquaculture species for many countries, also exhibits increased otolith growth rates in response to low pH during larval development. Our results agree with those of Réveillac *et al*. (2015), who reported that the otolith CaCO_3_ accretion rates in gilthead sea bream juveniles were increased in response to low pH ranging from 7.0 to 7.5^43^. No mechanism has been formally proposed to explain the increase in otolith growth rate in response to low pH. One hypothesis could be that elevated *p*CO_2_ and HCO_3_^-^ levels in the endolymph surrounding the otoliths would increase in parallel with plasma levels^53,54^ in response to low environmental pH, increasing otolith growth rates.

This is the first time that calcite deposition in otoliths has been observed in response to low pH. While calcite can be commonly found in exoskeletons and shells of marine invertebrates (e.g. bivalves)^55^, it is rarely found in teleost fish^56,57^ and is restricted to some non-teleost species in combination with vaterite^21^. The third known CaCO_3_ polymorph, vaterite, is commonly found in teleost fish reared under aquaculture conditions^22–26^ and a very recent meta-analysis has suggested that the deposition of vaterite might occur due to the fast growth rates achieved in the aquaculture industry^27^. Since we have not found evidence of different growth rates between treatments, it is unlikely that increased growth rates could be responsible for calcite deposition. Additionally, the results from the pedigree reconstruction revealed that the majority of half-sib families with more than 3 offspring comprised individuals from the three different treatments and showed calcitic and aragonitic otoliths. This indicates that a similar set of parents contributed in both spawning events and, therefore, the calcitic phenotype is not the result of an egg batch effect.

We used the narrow-sense heritability (*h*^2^) estimate as a proxy to evaluate the importance of genetic factors in calcite deposition due to the impossibility of measuring gene expression or determine genetic variants of genes involved in otolith formation in the current experimental setup. The *h*^2^ is the coefficient between additive genetic variance (∂_A_^2^, variance due to additive genetic effects) and the total phenotypic variance (∂_P_^2^, which includes environmental and phenotypic variance). Heritability estimates range between 1 (all the variance is explained by additive genetic effects) and 0 (all the variance is explained by other factors such as the environment, dominance and epistasis). The estimated *h*^2^ for calcitic otoliths (*h*^2^ = 0.44–0.55) suggests that a great proportion of the observed variability is explained by genetic factors, significantly higher than what is found in wild populations of animals (*h*^*2*^ = 0.1–0.2)^58^, but in line with traits measured in farmed fish^59^. Such high level of “influence” from genetic factors is not surprising if we consider that previous studies have demonstrated that the absence of structural genes required for otolith formation such as *otopetrin-1* (*otop1*) and *otolith matrix protein-1* (*omp-1*) can lead to the formation of calcitic otoliths^60,61^. Therefore, it is likely that behind the formation of calcitic otoliths might be alterations in gene expression or gene variants essential for otolith formation (structural proteins but also others such as HCO_3_^-^ transporters, etc). This could induce, for example, an impaired regulation of the endolymph pH and HCO_3_^-^ homeostasis (alkaline environments promote calcite deposition)^62^ or alterations in the otolith matrix proteins, both mechanisms have been suggested to favour calcite deposition^60,63,64^.

Despite some limitations of the present data, the significant proportion (21% on average) of individuals crystallising calcite at pH7.3 compared to pH8.2 and pH7.7 (where a small proportion of calcitic otoliths were also found) suggests that calcite deposition may be triggered by environmental pH, with small effects at pH7.7 (1–3% of animals affected) and exacerbated at pH7.3. It still remains to be elucidated whether calcitic otoliths could occur as a consequence of a pH-conditional phenotype (i.e. certain genetic variants would deposit aragonite under “permissive” environmental conditions whereas at a “restrictive” low pH would deposit calcite) or an epigenetic phenotype (i.e. low pH could modify epigenetic signals resulting in altered gene expression that would trigger calcite deposition).

While the number of studies reporting an increase in otolith growth rates in response to low pH is quite significant, there is very little research on how changes in otolith growth rates caused by ocean acidification will affect hearing, navigation and balance in fish. It has traditionally been suggested that otolith size and shape (especially in the case of the sagittae) are adaptive traits to different habitats and ecological niches, and that greater otolith mass would enhance hearing capacity^65,66^. However, this hypothesis has recently been questioned with studies that found no correlation between otolith size and hearing capacity for some groups of teleosts such as ophidiiformes^41^ and cichlids^67^. Simulations run by Bignami *et al*. (2013) found that bigger otoliths resulting from exposure to low pH have subtle differences in sound transmission, but they did not establish whether these variations translated in improved hearing capacities or not^39^. Recent studies have demonstrated that otolith composition might also affect fish hearing. Reimer *et al*. (2016) have found that salmonid otoliths that were mostly composed of vaterite had a 28–50% loss of otolith functionality^68^. Despite not having evaluated the sound conductivity of the otoliths, we believe it is possible that overgrown calcitic otoliths might also have a large impact on animal hearing. Perhaps such effects would be exacerbated if a synergy between size and composition exists. Further, extensive research is needed in order to confirm this hypothesis.

## Conclusions

The present work demonstrates, for the first time, that a significant proportion of gilthead sea bream larvae (21%) exposed to pH7.3 formed calcite as the main calcium carbonate polymorph for otolith formation instead of aragonite. The calcitic phenotype is likely the result of both environmental and genetic factors as suggested by an estimated *h*^*2*^ of 0.45–0.55. We also observed an increase in otolith growth rate in gilthead sea bream larvae when exposed to pH7.7 and pH7.3 after 18 days post-fertilization compared to controls at pH8.2. Further research is necessary to determine the impact of overgrown calcitic otoliths on fish hearing, orientation and balance.

## Electronic supplementary material

Supplementary Info
