# Supplementary material for: Ocean acidification promotes otolith growth and calcite deposition in gilthead sea bream (Sparus aurata) larvae
Source: Sci Rep. 2018 May 30;8:8384. doi: 10.1038/s41598-018-26026-y (PMC5976741; doi:10.1038/s41598-018-26026-y)
Supplement: Supplementary file 1 — Supplementary Info [file 41598_2018_26026_MOESM1_ESM.pdf]

Ocean acidification promotes otolith growth and calcite deposition in gilthead sea bream (*Sparus aurata*) larvae

Clara Coll-Lladó[a,b], Jan Giebichenstein[c], Paul B Webb[d], Christopher R Bridges[c] & Daniel Garcia de la serrana[a*].

[a]Gatty Marine Laboratory, Scottish Oceans Institute, School of Biology, University of St Andrews, Scotland (UK)

[b]Xelect Ltd, Horizon House, St Andrews, Scotland (UK)

[c]Institut für Stoffwechselphysiologie/AG Ecophysiology, Heinrich-Heine-Universität Düsseldorf, Germany

[d]School of Chemistry, University of St Andrews, Scotland (UK)

[*]Corresponding author: dgdlsc@st-andrews.ac.uk

Keywords: otolith, ocean acidification, pH, gilthead sea bream, calcite

Supplementary Figure 1

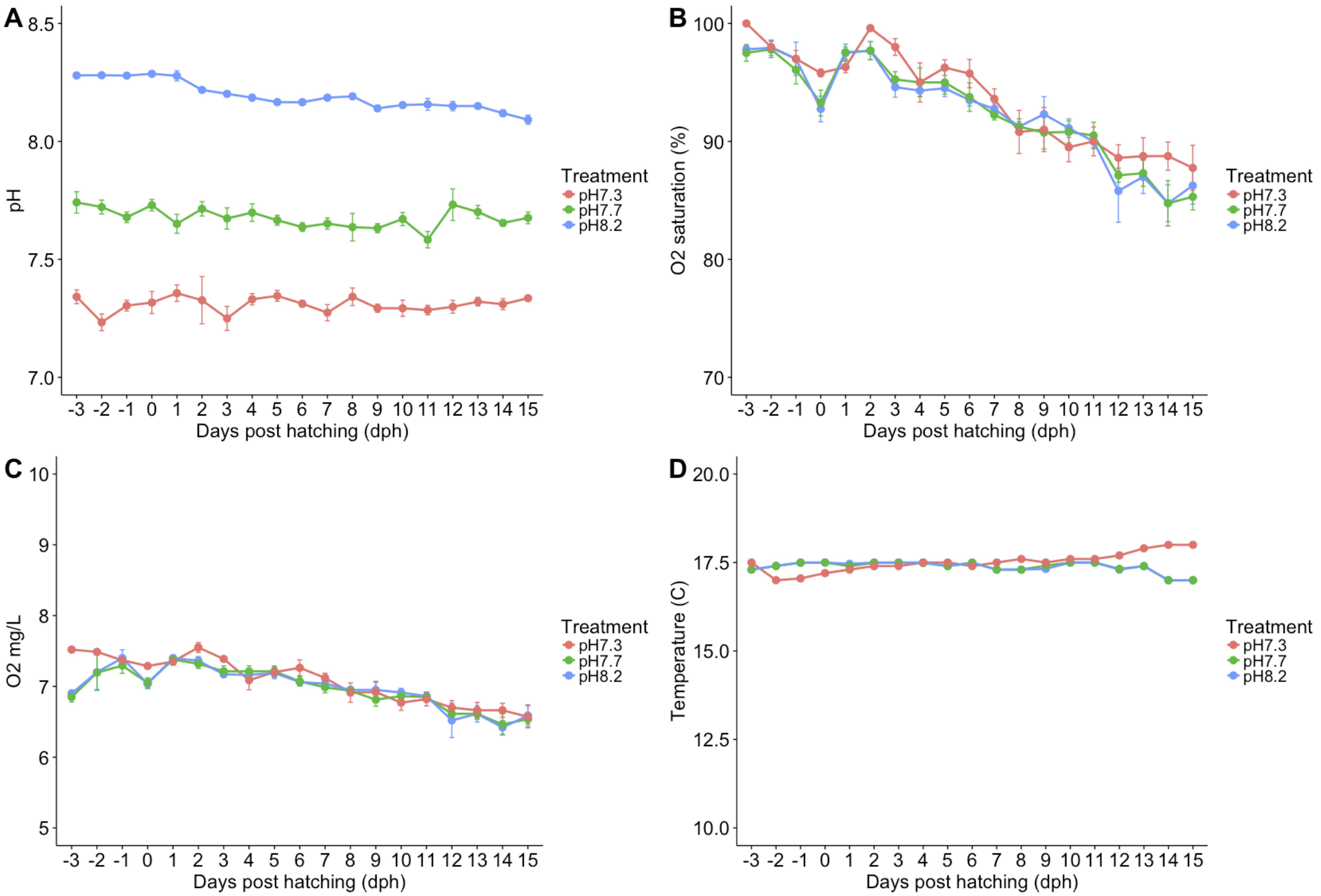

Supplementary Figure 1. Water parameters.

Water pH (**A**), oxygen saturation (**B**), dissolved oxygen (**C**) and temperature (**D**) during the experimental period for pH8.2 (blue line), pH7.7 (green line) and pH7.3 (red line) treatments.

Values represent the average±SD of 8 replicate tanks.

Supplementary Figure 2

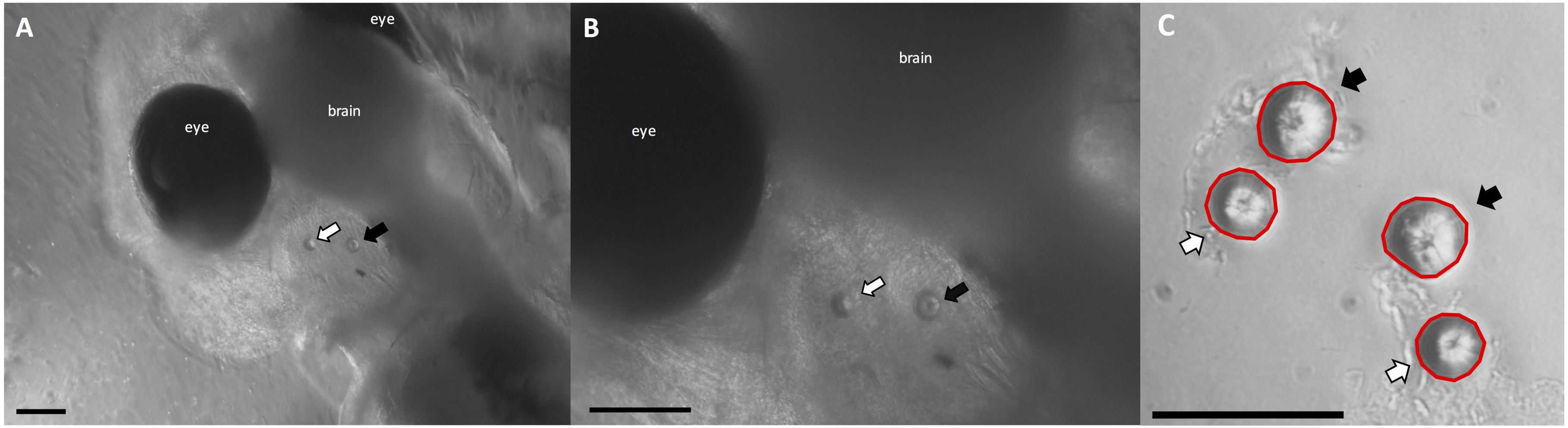

Supplementary Figure 2. Otolith location in gilthead sea bream larvae.
In situ location of the otoliths (arrows) in the head of a gilthead sea bream larvae (**A**). Detail of the gilthead sea bream larval otoliths (**B**). Magnified view of otoliths showing how otolith area and perimeter were measured (**C**).
The larger sagitta (black arrow) and the smaller lapillus (white arrow) are indicated. Scale bar indicates 100μm. An example of otolith measurement is shown as a red line in picture C.

Supplementary Figure 3

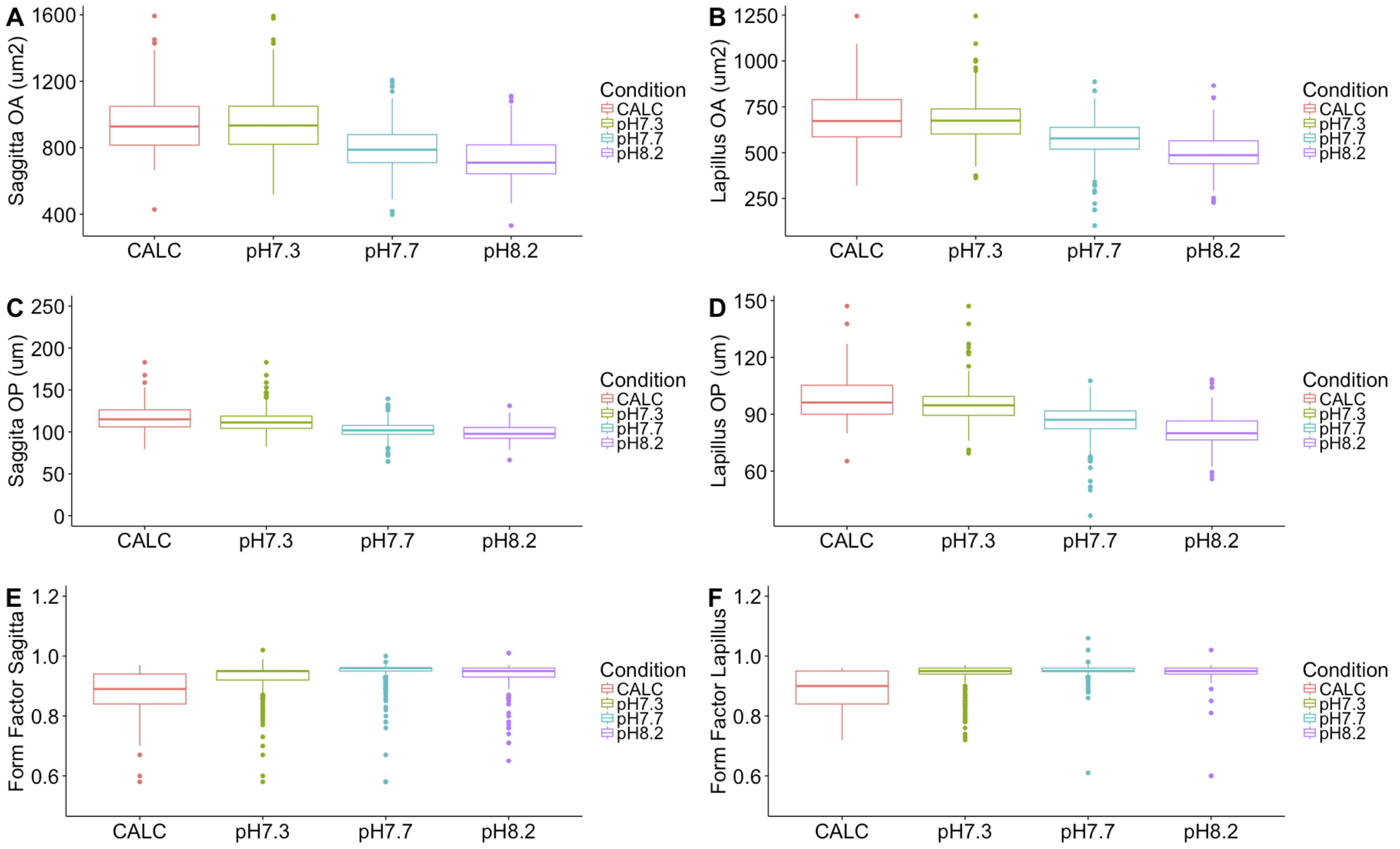

Supplementary Figure 3. Normalized OA, OP and Form Factor of calcitic otoliths.

Sagittae OA (**A**), lapilli OA (**B**), sagittae OP (**C**), lapilli OP (**D**), sagittae FF (**E**) and lapilli FF (**F**) for calcitic otoliths (red) compared to aragonitic otoliths from pH7.3 (green), pH7.7 (blue) and pH8.2 (purple) groups.

OA= otolith area; OP= otolith perimeter; FF= form factor; CALC=calcite

Supplementary Figure 4

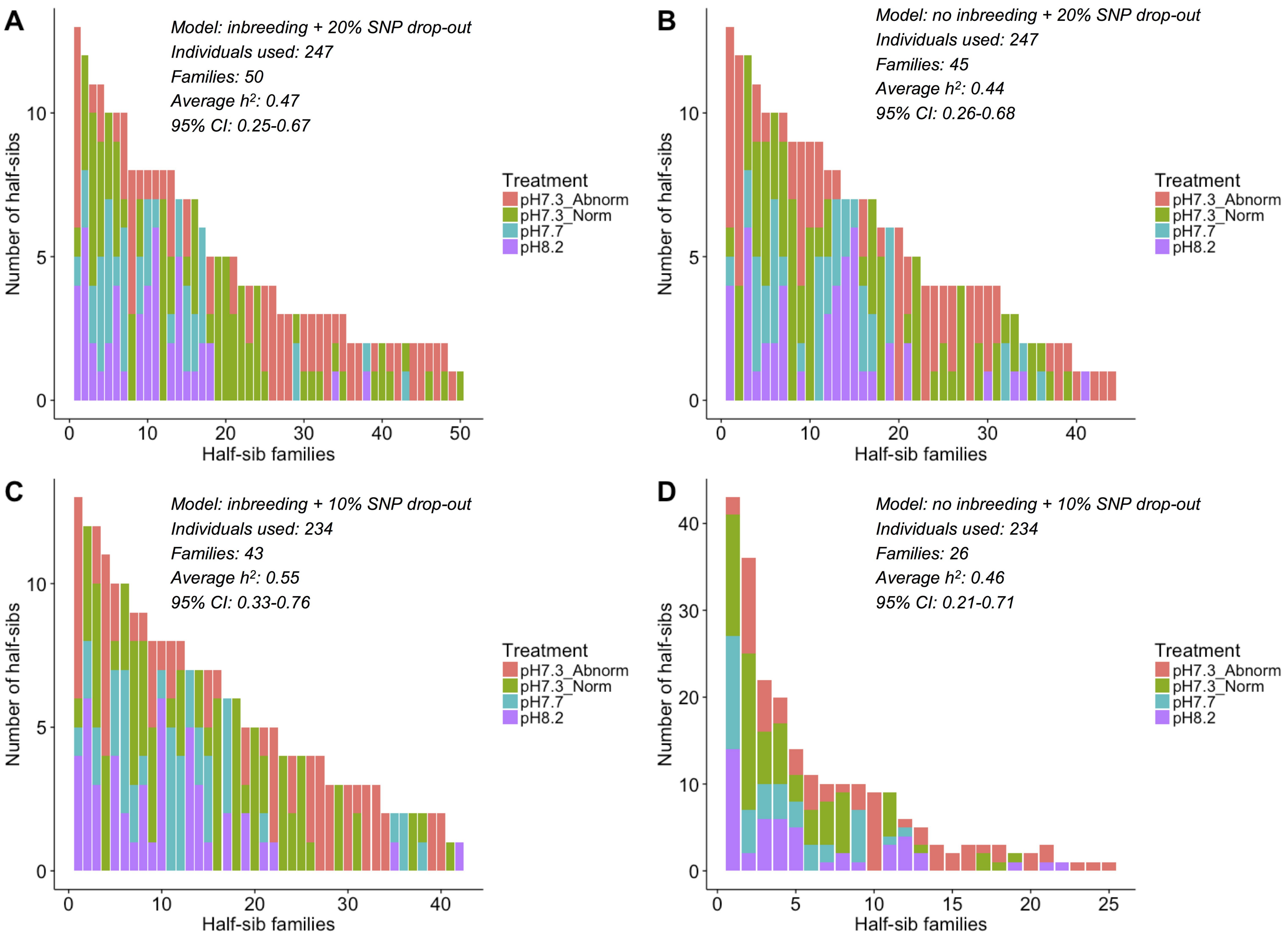

Supplementary Figure 4. Pedigree reconstruction models for gilthead sea bream larvae.
Family reconstruction for the larvae exposed to pH8.2, pH7.7 and pH7.3 (showing round and irregular otoliths) considering: (**A**) inbreeding and 20% SNP dropout (**B**) no inbreeding and 20% SNP dropout (**C**) inbreeding and 10% SNP dropout and (**D**) no inbreeding and 10% SNP dropout. Individual bars represent a half-sib family with the contribution of individuals from each treatment indicated in different colours. 95% CI= 95% confidence interval.
h^2= narrow-sense heritability.
